# β_2_-Agonist Induced cAMP Is Decreased in Asthmatic Airway Smooth Muscle Due to Increased PDE4D

**DOI:** 10.1371/journal.pone.0020000

**Published:** 2011-05-17

**Authors:** Thomas Trian, Janette K. Burgess, Kyoko Niimi, Lyn M. Moir, Qi Ge, Patrick Berger, Stephen B. Liggett, Judith L. Black, Brian G. Oliver

**Affiliations:** 1 Cell Biology, Woolcock Institute of Medical Research, Sydney, New South Wales, Australia; 2 Discipline of Pharmacology, School of Medical Sciences, The University of Sydney, Sydney, New South Wales, Australia; 3 Centre de Recherce Cardio-Thoracique de Bordeaux, Université Bordeaux Segalen, INSERM, Bordeaux, France; 4 Department of Medicine, University of Maryland School of Medicine, Baltimore, Maryland, United States of America; University of Giessen Lung Center, Germany

## Abstract

**Background and Objective:**

Asthma is associated with airway narrowing in response to bronchoconstricting stimuli and increased airway smooth muscle (ASM) mass. In addition, some studies have suggested impaired β-agonist induced ASM relaxation in asthmatics, but the mechanism is not known.

**Objective:**

To characterize the potential defect in β-agonist induced cAMP in ASM derived from asthmatic in comparison to non-asthmatic subjects and to investigate its mechanism.

**Methods:**

We examined β_2_-adrenergic (β_2_AR) receptor expression and basal β-agonist and forskolin (direct activator of adenylyl cyclase) stimulated cAMP production in asthmatic cultured ASM (n = 15) and non-asthmatic ASM (n = 22). Based on these results, PDE activity, PDE4D expression and cell proliferation were determined.

**Results:**

In the presence of IBMX, a pan PDE inhibitor, asthmatic ASM had ∼50% lower cAMP production in response to isoproterenol, albuterol, formoterol, and forskolin compared to non-asthmatic ASM. However when PDE4 was specifically inhibited, cAMP production by the agonists and forskolin was normalized in asthmatic ASM. We then measured the amount and activity of PDE4, and found ∼2-fold greater expression and activity in asthmatic ASM compared to non-asthmatic ASM. Furthermore, inhibition of PDE4 reduced asthmatic ASM proliferation but not that of non-asthmatic ASM.

**Conclusion:**

Decreased β-agonist induced cAMP in ASM from asthmatics results from enhanced degradation due to increased PDE4D expression. Clinical manifestations of this dysregulation would be suboptimal β-agonist-mediated bronchodilation and possibly reduced control over increasing ASM mass. These phenotypes appear to be “hard-wired” into ASM from asthmatics, as they do not require an inflammatory environment in culture to be observed.

## Introduction

Clinically, asthma is characterized by variable degrees of obstruction due to active airway smooth muscle (ASM) contraction, increased ASM mass, and increased airway mucus. Some studies have also suggested that asthmatics have decreased β_2_AR function compared to normal subjects [1], [2], which is also supported by animal and *ex vivo* studies [3], [4], [5], [6], [7]. While it is now accepted that ASM cells play a central role in the pathophysiology of asthma [8], [9], [10], the mechanisms of the various phenotypes remain somewhat ill-defined. We and others have previously reported that calcium (Ca^2+^) homeostasis is altered in asthmatic ASM cells [11], [12]. The dysregulation of this second messenger is associated with an increase in asthmatic ASM cell proliferation that underlies the ASM remodelling which accompanies asthma [12], [13], and accounts for the readily contracted ASM in asthmatics upon exposure to agents such as methacholine. Cyclic adenosine monophosphate (cAMP) is another important regulatory second messenger, which mediates ASM cell relaxation and inhibition of proliferation. cAMP production in ASM cells can be caused by any of a number of expressed G-protein coupled receptors (GPCRs) that couple to G_αs_ which stimulates adenylyl cyclase (AC). Therapeutically, β-agonists acting on β_2_AR expressed on ASM are responsible for cAMP production and the clinical effects. This pathway has been extensively dissected in cells which have been recombinantly manipulated, and endogenously expressing cells including human ASM. Nevertheless, mechanisms of β_2_AR dysfunction in asthma have been hampered by the potentially confounding effects of β-agonists and corticosteroids when using freshly obtained airway tissue. [2], [14], [15], [16]. In vitro chronic treatment with β_2_-agonists such as metaproterenol and albuterol induced a reduction of membrane β_2_AR expression by internalisation of the receptor [15], [16]. Autoradiographic enumeration of the β_2_AR has shown no decrease in asthmatic tissue compare to non asthmatic [14], however bronchial tissue from asthmatic patients are less responsive to β_2_-agonists in comparison to non-asthmatics [2]. Additionally when primary ASM cell lines are cultured the notion that mimicking the inflammatory milieu is necessary has also potentially confounded research in this area. β_2_AR is the most utilized target of asthma therapeutics, both for rescue and prevention, with the use of both short- and long-acting β-agonists. An additional response to β-agonists is a reduction in human ASM cell proliferation [13], that is also cAMP dependent. Thus, cAMP seems to be involved in several pivotal events in ASM pathophysiology but its production and regulation have not been directly investigated in ASM from asthmatic patients.

Phosphodiesterases (PDE) act as key regulators of this pathway by degrading cyclic nucleotides. PDEs represent a super family of enzymes divided into 11 subfamilies that can degrade cytosolic cAMP and cAMP. In human ASM cells, PDE4 has been found to be the major PDE subtype that is involved in cAMP degradation [17] and PDE4 inhibitors such as roflumilast and cilomilast have been developed recently, in particular for the treatment of COPD.

We hypothesised that increased PDE4D in asthmatic airway smooth muscle was contributing to the β_2_AR “defect” observed in asthma. Using ASM cells from normal and asthmatic subjects β_2_-agonist induced cAMP production was assessed. A profound decrease in β-agonist stimulated cAMP was found, which led to additional studies indicating a post-receptor phenomenon. Increased PDE activity from increased PDE4D expression in asthmatic ASM was found. Clinically studied PDE4 inhibitors eliminated the decrease in β-agonist signalling to cAMP as well as cell proliferation.

## Materials and Methods

### Ethics Statement

The study was approved by the Ethics Review Committee of the Sydney South West Area Health Service, Royal Prince Alfred Hospital and The University of Sydney human research ethics committee. All volunteers provided written informed consent.

We established primary cell cultures of human ASM cells from explants of ASM bundles. These were obtained from macroscopically normal surgical specimens following resection surgery for thoracic lesions or lung transplantation, or from airway biopsies as previously described [18]. Subject details are shown in Table S1. Asthma was defined by GINA guidelines [19]. Human ASM cells were maintained in DMEM medium supplemented with 10% FBS in 5% CO_2_, 95% air incubator at 37°C. No other supplements (such as antibiotics or pro-inflammatory agents) were utilized during experimentation. ASM characterization was confirmed by means of light microscopy and immunohistochemistry for smooth muscle α-actin and calponin expression. Cells were studied at 80% confluence between passages five and eight. From the time of collection from the subjects to the first studies was typically at least four weeks in culture.

cAMP production was assessed by stimulating 5×10^3^ ASM cells for 5 minutes with log increasing concentrations of the β-agonists isoproterenol from 10^−9^ M to 10^−5^ M, albuterol (10^−8^ M and 10^−7^ M) and formoterol (10^−8^ M and 10^−7^ M). Measurements of intracellular cAMP concentration were performed using the alpha screen cAMP kit (Perkin Elmer, Melbourne, Australia) as previously described [10]. In some experiments cAMP production was also measured after stimulation with forskolin (10 µM) and incubation with PDE inhibitors. The pan-PDE inhibitor 3-Isobutyl-1-methylxanthine (IBMX) was used at 0.5 mM [20]. The PDE4 inhibitors roflumilast 1 µM (Nycomed) and cilomilast 1 µM (GSK) and the PDE3 inhibitor milrinone (10 µM, Sigma-Aldrich, Castle Hill, NSW) were utilized at the indicated concentrations based on reports showing complete and selective inhibition of their respective isoenzymes [20], [21], [22], [23], [24].

The level of β_2_AR expression was assessed using flow cytometry as previously described [10]. Briefly, fixed HASMC (80–90% confluent), were blocked for 1 hour with 4% BSA in PBS, incubated for 1 hour on ice with the primary antibody (rabbit anti-human β_2_-adrenergic receptor, Abcam, San Francisco, USA), washed twice in PBS and then incubated with the secondary antibody (Alexa Fluor 488-conjugated goat anti-rabbit antibody; Molecular Probes, Carlsbad, USA). An appropriate isotype control was purchased from Becton Dickinson. Cells were then washed again and resuspended in PBS prior to FACS analysis. Analysis was conducted on Becton Dickinson FACSCalibur Sort using CellQuest Software. Events were gated on forward and side scatter parameters to include only live cells. Changes in fluorescence (i.e., receptor expression) were determined by measuring median fluorescence of positive cells.

PDE3A, 4D and 5A expression in non-asthmatic and asthmatic cells was assessed by western blotting using primary antibodies directed against PDE3A, PDE4D and PDE5A. Briefly, whole lysates from ASM cells were collected using 1% Triton X-100 lysis buffer in the presence of 2 mM sodium orthovanadate, 1 mM EDTA, 50 µg/ml aprotinin, 100 µM leupeptin, 1 mM 1,4-Dithio-DL-Treitol, and 1 mM amino-ethyl-benzenesulfonyl fluoride hydrochloride (all from Sigma-Aldrich). Cellular extracts were reduced with β-mercaptoethanol, subjected to electrophoresis on a 10% acrylamide reducing gel, and transferred to PVDF membrane (Immobilon TM-P; Millipore). The immunoblots were then developed using rabbit anti–human PDE4D (Santa Cruz, Santa Cruz, USA), rabbit anti–human PDE3A (Santa Cruz), rabbit anti–human PDE5A (Santa Cruz) or mouse anti–human GAPDH (Chemicon, Billerica, USA) overnight at 4°C. For amplification, a biotinylated goat secondary anti–mouse IgG (Bio-Rad Laboratories) or anti–rabbit IgG (Santa Cruz Biotechnology, Inc.) was added for 2 h at room temperature followed by a streptavidin horseradish peroxidase complex (Dako Carpinteria, USA). Immunoblots were revealed by enhanced chemiluminescence (Uptima, Montluçon, France). Blot images were acquired using BioCaptMW (Thermo Fisher Scientific, Pittsburgh, USA), and band densities were quantified using Image J software.

PDE activity was assayed in human ASM cell lysates using a colorimetric assay method from Biomol (Enzo life science, Plymouth Meeting, USA), following the manufacturer's protocol. The measured levels of PDE activity were standardized to protein content in the cell extracts to express the result as an optical density value divided by protein concentration of the cell lysate.

For measurement of ASM cellular proliferation, 5×10^4^ ASM cells per well were seeded in a 6 well plate and then quiesced with 1% BSA in DMEM medium for 48 hours. Then, proliferation of the cells was stimulated by replacing the medium with DMEM supplemented with 10% FBS. The PDE inhibitors (IBMX 0.5 mM; roflumilast 1 µM and cilomilast 1 µM) were also added at the time of stimulation. After 3 days, cells were harvested by trypsin and counted using a hematocytometer, with at least 500 cells counted within the grid.

Comparisons of the data were carried out by ANOVA with Dunnett's post test. Post hoc tests were carried out when p<0.05 by ANOVA. Data were analysed using GraphPad Prism version 5.00 for Windows (GraphPad Software, San Diego, California, USA). A probability level of 95% (*P*≤0.05) was considered as the threshold for statistical significance. Data are shown as mean±SEM.

## Results

**Figure 1:**
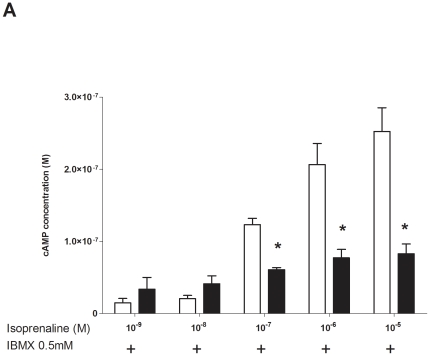
Isoproterenol induced cAMP in ASM cells from asthmatic and non-asthmatic patients. cAMP degradation was inhibited by addition of a pan-PDE inhibitor IBMX 0.5 mM (n = 15 non asthmatic and n = 10 asthmatic). Results are presented as mean ± SEM. * denotes a significant difference from non-asthmatic (*P*≤0.05).

Human ASM cells from asthmatic and non-asthmatic volunteers were stimulated with the β-agonist isoproterenol and cAMP levels measured. Since the measurement of cAMP in these cells is only possible in the presence of PDE inhibitors such as IBMX that prevent cAMP degradation, in these initial cAMP experiments, IBMX was used at the standard concentration of 0.5 mM (Fig. 1A). These results revealed markedly lower cAMP levels in asthmatic ASM, which were statistically different at 10^−7^ to 10^−5^ M, but a trend was observed at all doses. A similar loss-of-function was observed when the β-agonists albuterol and formoterol were utilized (Fig S1).

We excluded the possibility that the difference was due to differential β_2_AR expression on the ASM cell surface. Using flow cytometry, we found no differences in β_2_AR expression between asthmatic and non-asthmatic ASM cells (Fig S2A, B). Moreover, there was no difference in the basal cAMP level in the absence of β-agonist between the two cell groups (Fig S2C). We also assessed cAMP production independent of the β_2_AR using stimulation of AC with forskolin. As before, we found decreased cAMP production in asthmatic compared with non-asthmatic ASM (Fig S2C).

Taken together, these results suggest that neither β_2_AR expression, nor its coupling to AC can account for the decreased β-agonist promoted cAMP stimulation. Rather, a post-AC defect was suggested, particularly in the degradation of cAMP by PDE.

**Figure 2:**
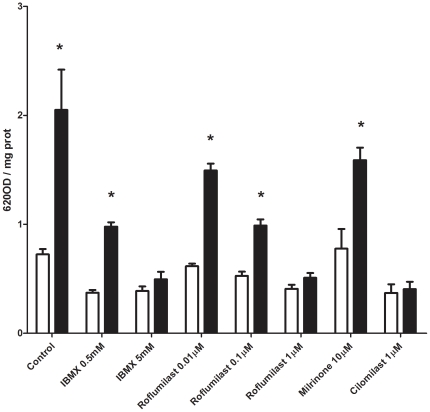
PDE activity is increased in asthmatic ASM cells. PDE activity was assayed in human ASM cell lysates using a colorimetric assay kit (n = 4 non-asthmatic and n = 4 asthmatic). Cell lysates derived from non-asthmatic and asthmatic patients were used in the absence (Control) or presence of PDE inhibitors (IBMX 0.5 mM and 5 mM; roflumilast 0.01 µM, 0.1 µM and 1 µM, milrinone 10 µM and cilomilast 1 µM). Data represent mean ± SEM of the OD activity value divided by protein concentration of the cell lysate. * Denotes a significant difference from control (*P*≤0.05).

To confirm this hypothesis, we assessed PDE activity in asthmatic and non-asthmatic ASM cells. Following isolation of cellular proteins, we found that total PDE activity was higher (∼2 fold) in asthmatic ASM cells compared to ASM cells derived from non-asthmatic patients (Fig. 2). This is consistent with our findings with regard to cAMP levels and PDE inhibition (Fig. 1). In the non-asthmatic ASM cells IBMX at 0.5 mM reduced PDE activity and this was not further reduced when the concentration was increased to 5.0 mM, however in the asthmatic cells, further reduction occurred (IBMX 0.5 mM = 60% of effect of IBMX 5.0 mM, Fig. 2). To establish that PDE4 was the major PDE responsible for degradation of cAMP, we independently utilized IBMX or the specific PDE4 inhibitors roflumilast (1 µM, 0.1 µM and 0.01 µM) and cilomilast (1 µM). Treatment of the asthmatic cells with the specific PDE4 inhibitors resulted in a marked increase in the amount of cAMP measured, suggesting that the cAMP PDE-cAMP activity is predominantly dependent on PDE4 in ASM, and 0.5 mM IBMX is not sufficient to inhibit all PDE activity from the asthmatic cells.

**Figure 3:**
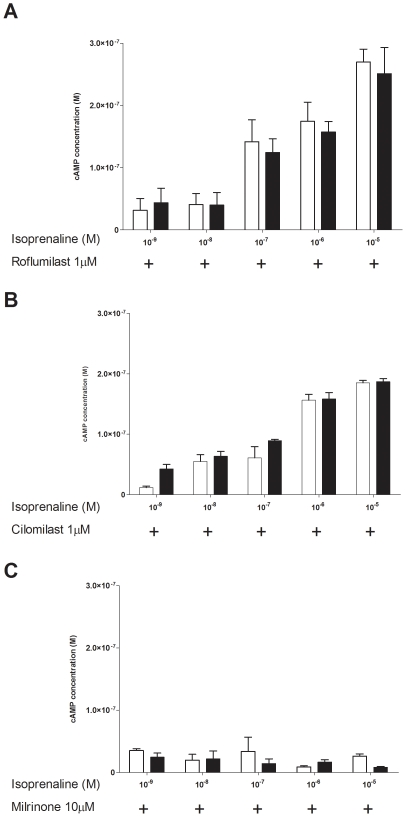
PDE4-specific inhibitors normalize asthmatic ASM cAMP production. Isoproterenol (10^−9^ M to 10^−5^ M) induced cAMP in ASM cells from asthmatic and non-asthmatic patients. cAMP degradation was inhibited by addition of different PDE inhibitors, A: roflumilast 1 µM (n = 7 non-asthmatic and n = 5 asthmatic); B: cilomilast 1 µM (n = 5 non-asthmatic and n = 5 asthmatic) and C: milrinone 10 µM (n = 3 non-asthmatic and n = 3 asthmatic). Results are presented as mean ± SEM.

We then confirmed the involvement and specificity of PDE4 in ASM cells by comparing the two specific PDE4 inhibitors with a PDE3 inhibitor (milrinone) at standard concentrations known to completely and selectively inhibite their respective isoenzyme [24]. We found no difference in β-agonist-induced cAMP production in cells derived from asthmatic and non-asthmatic patients in the presence of the two specific PDE4 inhibitors roflumilast and cilomilast (Fig. 3A and 3B). As previously described [25] we also confirmed that PDE3 was not involved in the degradation of cAMP induced by β-agonists in ASM cells (Fig. 3C). This result confirms the major role of PDE4 in ASM cells and suggests that asthmatic cells specifically have greater PDE4 activities compared to control cells.

**Figure 4:**
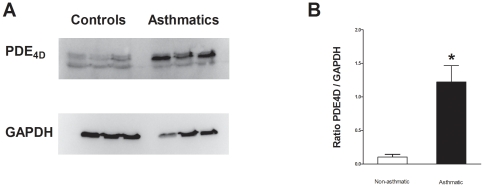
Enhanced PDE4D in asthmatic ASM. Western blot analysis of PDE4D. A: represents a typical western blot for PDE4D. The bands on the left are from 3 non asthmatic, and on the right from 3 asthmatic patients. B: represents PDE4/GAPDH ratio mean ± SEM (n = 8 non-asthmatic and n = 8 asthmatic). * Denotes a significant difference from non-asthmatic (*P*≤0.05).

Since PDE4D has previously been shown to be the specific PDE4 subtype involved in the modulation of cAMP production in ASM cells [25], we tested the hypothesis that the enhanced activity is due to increased protein expression by measuring the amount of PDE4D by western blots, and found significantly increased expression in ASM cells from patients with asthma compared to patients without asthma (Fig. 4A and B). To investigate if other PDEs were also upregulated in the asthmatic cells we measured the expression of PDE3A and PDE5A. As shown in Figures S3A and S3B, we found no differences in the expression of those two PDE subtypes between asthmatic and non-asthmatic ASM cells.

**Figure 5:**
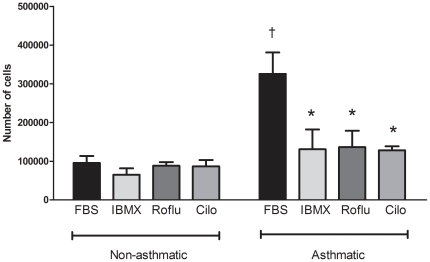
Cell proliferation phenotypes differ between normal and asthmatic ASM. 5×10^4^ asthmatic (n = 4) and non-asthmatic (n = 4) ASM cells per well were seeded and incubated with DMEM supplemented with FBS 10% only (black bars) or with 10% FBS in DMEM and the following PDE inhibitors IBMX (0.5 mM), roflumilast (roflu, 1 µM) and cilomilast (cilo, 1 µM). ASM cells were counted after 3 days growth. Data represent mean ± SEM of the number of cells after 3 days. * denotes a significant difference from FBS and † denotes a significant difference from non-asthmatic (*P*≤0.05).

As asthmatic ASM cells are known to have a greater proliferation rate than non-asthmatics, and β-agonists/cAMP inhibit ASM proliferation [26], we also assessed if the altered PDE4 expression in asthmatic ASM cells affected proliferation. As shown in Fig. 5, asthmatic ASM cells proliferate at a greater rate than non-asthmatic cells. When the PDE inhibitor IBMX or the two specific PDE4 inhibitors roflumilast and cilomilast were used, the proliferation of the asthmatic cells was significantly decreased, however there was no effect on the proliferation rate of the non-asthmatic cells (Fig. 5).

## Discussion

The body of evidence for an intrinsic abnormality in ASM and its signalling characteristics in asthma is increasing rapidly. This study highlights, another particularly relevant difference between the cellular properties of asthmatic and non-asthmatic ASM. Our findings show that stimulation of the β_2_AR either by isoproterenol or two clinically utilized β-agonists, albuterol and formoterol, results in less cAMP production in asthmatic cells. β_2_AR expression was not different between the two sets of cells and forskolin-stimulated activities were also less in the asthmatic ASM cells. These results pointed towards a post-receptor, post-AC defect, and the use of isotype-specific PDE inhibitors revealed a normalization of β-agonist stimulated cAMP levels. This result can be explained by the increased activity of PDE in asthmatic ASM cells. These results suggest that there is an increased activity of PDE in asthmatic ASM cells, and, that this can be attributed to an increase in the activity of PDE4. Moreover, we found that asthmatic ASM cells overexpress the PDE4D isoform when compared to non-asthmatic cells. As previously described by Mehats et al, we found that PDE4 is the major PDE subtype responsible for the degradation of cAMP in the ASM cell [25].

A potential defect in βAR signalling in asthmatics has been suggested by a number of human and animal model studies. In humans, these studies are particularly difficult since normal subjects show a minimal to no FEV-1 response to β-agonists, and in asthmatics the concurrent use of β-agonists or glucocorticoids can alter β_2_AR expression and/or function. An early study of extrapulmonary functions such as pupillary size suggested autonomic dysregulation in asthmatics that was consistent with β_2_AR dysfunction [1]. In *ex vivo* studies of asthmatic and non-asthmatic airways, the potency of β-agonists has been reported to be ∼5–10-fold less in asthmatic airways [2], which was not associated with a loss of β_2_AR expression [14]. In the ovalbumin-sensitized animal model of inflammation and airway hyperresponsiveness, β-agonist- promoted relaxation of methacholine constricted airways is decreased compared to that in control mice [3], which is also found in the IL-13 overexpression model [4]. Similar findings have been reported with animal or human airways exposed to various pro-inflammatory cytokines, prostanoids or allergic serum [5], [6], [7]. These latter studies potentially implicate locally generated factors from the inflammatory/asthmatic milieu in βAR hyperresponsiveness, but do not directly compare βAR function of ASM from non-asthmatic and asthmatic airways. As indicated earlier, such studies must consider the effects of treatment on β_2_AR expression and function. For example, in normal subjects treated with 6 inhalations of the β-agonist metaproterenol, in bronchoscopy-derived epithelial cells and macrophages there was an approximate 70% decrease in β_2_AR expression and 40–50% decrease in functional coupling to cAMP [15]. Profound desensitization from 6–12 hours of submicromolar concentrations of albuterol exposure [16] is also observed with human bronchi using precision cut lung slices. Such desensitization has been shown to be inhibited by glucocorticoids [16], [27].

We have considered, though, that ASM from asthmatics may have specific signalling characteristics due to genetic variation or epigenetic mechanisms, such that when maintained in standard culture media, differences between asthmatic and non-asthmatic ASM signalling will be apparent. Under these circumstances there would be no need for specialized media to mimic the asthmatic milieu, and drug effects from treatment of the asthmatics would not be an issue. As shown in the current study, β-agonist mediated cAMP production is indeed abnormal in asthmatic ASM, being substantially less than that in non-asthmatic ASM. Another apparent phenotype in asthmatic ASM is increased Ca^2+^ production from G_q_-coupled receptor signalling, as we and others have recently reported [11], [12]. The consequences of these aberrations, decreased β-agonist responsiveness and increased responsiveness to bronchospastic stimuli, are entirely consistent with the spectrum of clinical phenotypes observed in asthma. It is important to note, though, that we do not suggest a secondary role for inflammation in programming ASM pathobiology. Rather, we propose that some phenotypes can be observed in asthmatic ASM even in the absence of inflammation, pointing towards genetic or epigenetic predisposition. Furthermore, they are likely modified further by specific triggers, such as the β_2_AR dysfunction imposed by viral infection ref, crosstalk from other GPCRs, and inflammatory events. In terms of the current work, a component of this underlying predisposition for β_2_AR dysfunction may be amenable to therapeutic intervention with PDE4 inhibitors.

Indeed, Rabe et al demonstrated the benefit of roflumilast in COPD patients in a multicenter double blind randomized study of 24 weeks. They found an improvement in the postbronchodilator FEV-1 and in health-related quality of life in patients treated with this drug [28]. Moreover, another randomized, placebo-controlled, double-blind study was conducted two years later over 12 months by Calverley et al. In this extended study, an improvement of the postbronchodilator FEV-1 by roflumilast was also reported [29]. In asthma, roflumilast treatment has been shown to reduce airway hyperresponsiveness after histamine challenge in allergic patients with mild asthma [30] and to improve FEV-1, asthma symptom scores and rescue inhaler use in a 12 week randomized double blind clinical study [31].

Finally in our study, we found that the increase in PDE4 activity and expression may be involved in the hyperplasia of asthmatic ASM cells. As we have previously described [12], [18], [32], asthmatic ASM cells have a higher proliferation rate. In the present study, we found that in asthmatic ASM cells, three days of complete and selective PDE4 inhibition could reduce proliferation to a rate similar to that found in the non-asthmatic ASM cells. This finding is of clinical relevance, as ASM cell remodelling is likely to be involved in the decreased lung function associated with the disease, but remains insensitive to the usual asthma therapies [33]. Our finding may suggest, therefore, that PDE4 is a therapeutic target of interest in the area of ASM remodelling. The involvement of other PDEs in cell proliferation elsewhere has been shown previously. For example, a specific inhibitor of PDE7 decreased proliferation of murine natural killer T cells [34], and the PDE inhibitor pentoxifylline decreased proliferation of the rat epithelial cell line IEC18 [35].

In conclusion, this study demonstrates β_2_AR mediated cAMP generation is impaired in asthmatic ASM caused by enhanced cAMP degradation by PDE4, due to increased PDE4 expression. This signalling bias appears to be “hard-wired” in asthmatic ASM but can be mitigated by PDE4 inhibitors, suggesting additional studies on therapeutics aimed at this nodal point.

## Supporting Information

Figure S1 Isoproterenol (10^−6^ M and 10^−5^ M), albuterol (10^−8^ M and 10^−7^ M) and formoterol (10^−8^ M and 10^−7^ M) induced cAMP in ASM cells from asthmatic (n = 3) and non-asthmatic (n = 3) patients. cAMP degradation was inhibited by addition of 0.5 mM IBMX. Results are presented as mean ± SEM. * denotes a significant difference from asthmatic (*P*≤0.05).
(TIF)

Figure S2 ASM cell β_2_AR expression as assessed by flow cytometry. A: Representative flow cytometry data for membrane β_2_AR expression. ASM cells are stained with isotype control (black) or β_2_AR antibody (red and blue). ASM cells were obtained from non-asthmatic (red) and asthmatic (blue) patients. B: Data are presented as the mean values ± SEM for the median fluorescence of positive cells. ASM cells were obtained from non-asthmatic (white bar n = 5) and asthmatic (black bar n = 5) patients. NS = no significant difference. C: cAMP production in ASM cells from non-asthmatic (n = 4) and asthmatic (n = 5) patients. Cells are either unstimulated or stimulated with forskolin (10 µM). Results are presented as mean ± SEM. * denotes a significant difference from control (*P*≤0.05). NS = no significant difference.
(TIF)

Figure S3 Western blot analysis of A: PDE3B (n = 4 non-asthmatic and n = 4 asthmatic) and B: PDE5A (n = 4 non-asthmatic and n = 4 asthmatic) expression in non-asthmatic and asthmatic ASM cells. A typical western blot is shown for each PDE and the data are summarized in the graph as PDE/GAPDH ratio mean ± SEM. The bands on the left are from non asthmatic, and on the right from asthmatic patients. NS = no significant difference.
(TIF)

Table S1 Demographics of patient data.
(DOCX)
