# Supplementary figures and images for: β2-Agonist Induced cAMP Is Decreased in Asthmatic Airway Smooth Muscle Due to Increased PDE4D
Source: PLoS One. 2011 May 17;6(5):e20000. doi: 10.1371/journal.pone.0020000 (PMC3096656; doi:10.1371/journal.pone.0020000)

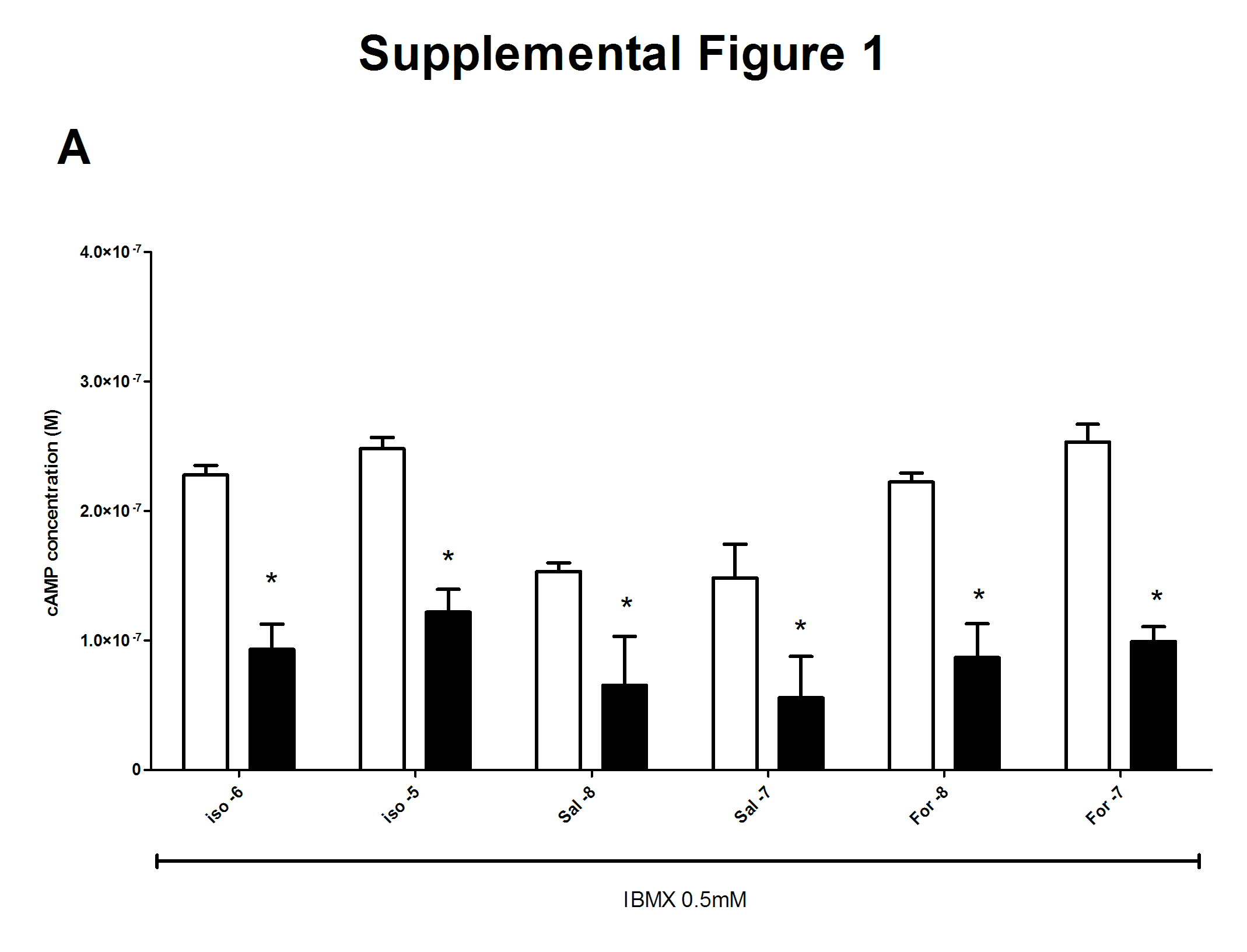

Supplement: Figure S1 — Isoproterenol (10−6 M and 10−5 M), albuterol (10−8 M and 10−7 M) and formoterol (10−8 M and 10−7 M) induced cAMP in ASM cells from asthmatic (n = 3) and non-asthmatic (n = 3) patients. cAMP degradation was inhibited by addition of 0.5 mM IBMX. Results are presented as mean ± SEM. * denotes a significant difference from asthmatic (P≤0.05). (TIF) [file pone.0020000.s001.tif]

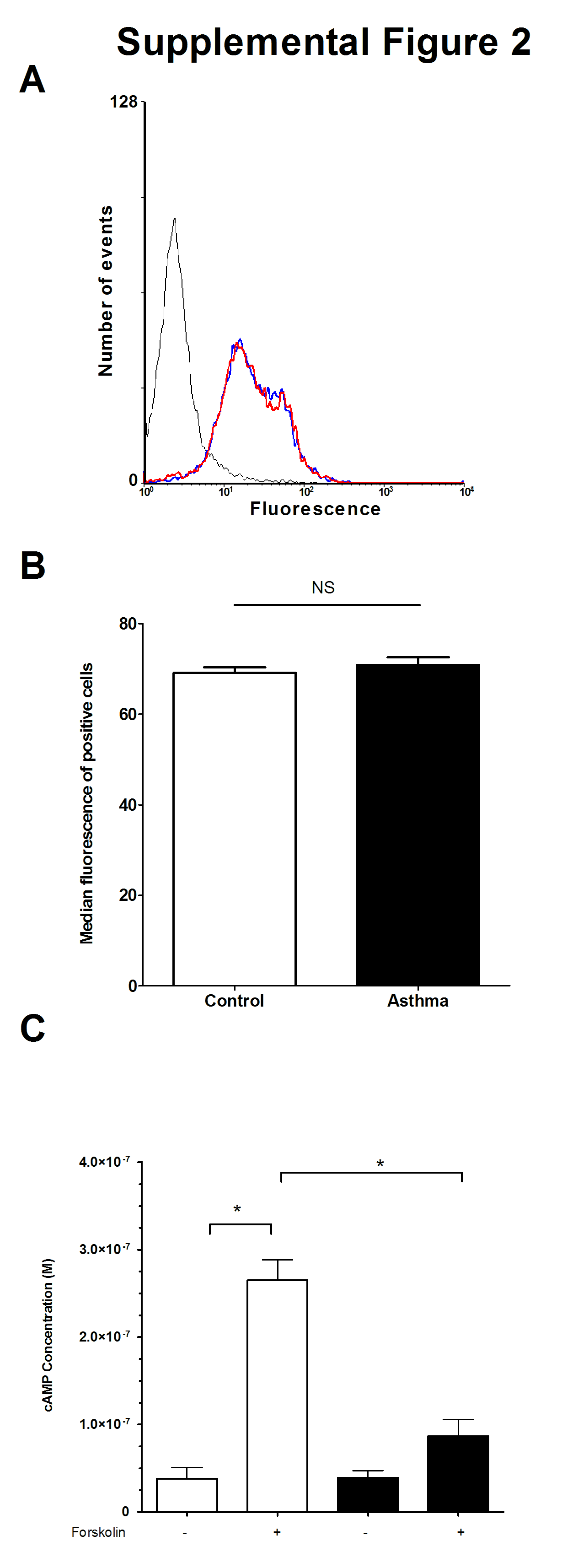

Supplement: Figure S2 — ASM cell β2AR expression as assessed by flow cytometry. A: Representative flow cytometry data for membrane β2AR expression. ASM cells are stained with isotype control (black) or β2AR antibody (red and blue). ASM cells were obtained from non-asthmatic (red) and asthmatic (blue) patients. B: Data are presented as the mean values ± SEM for the median fluorescence of positive cells. ASM cells were obtained from non-asthmatic (white bar n = 5) and asthmatic (black bar n = 5) patients. NS = no significant difference. C: cAMP production in ASM cells from non-asthmatic (n = 4) and asthmatic (n = 5) patients. Cells are either unstimulated or stimulated with forskolin (10 µM). Results are presented as mean ± SEM. * denotes a significant difference from control (P≤0.05). NS = no significant difference. (TIF) [file pone.0020000.s002.tif]

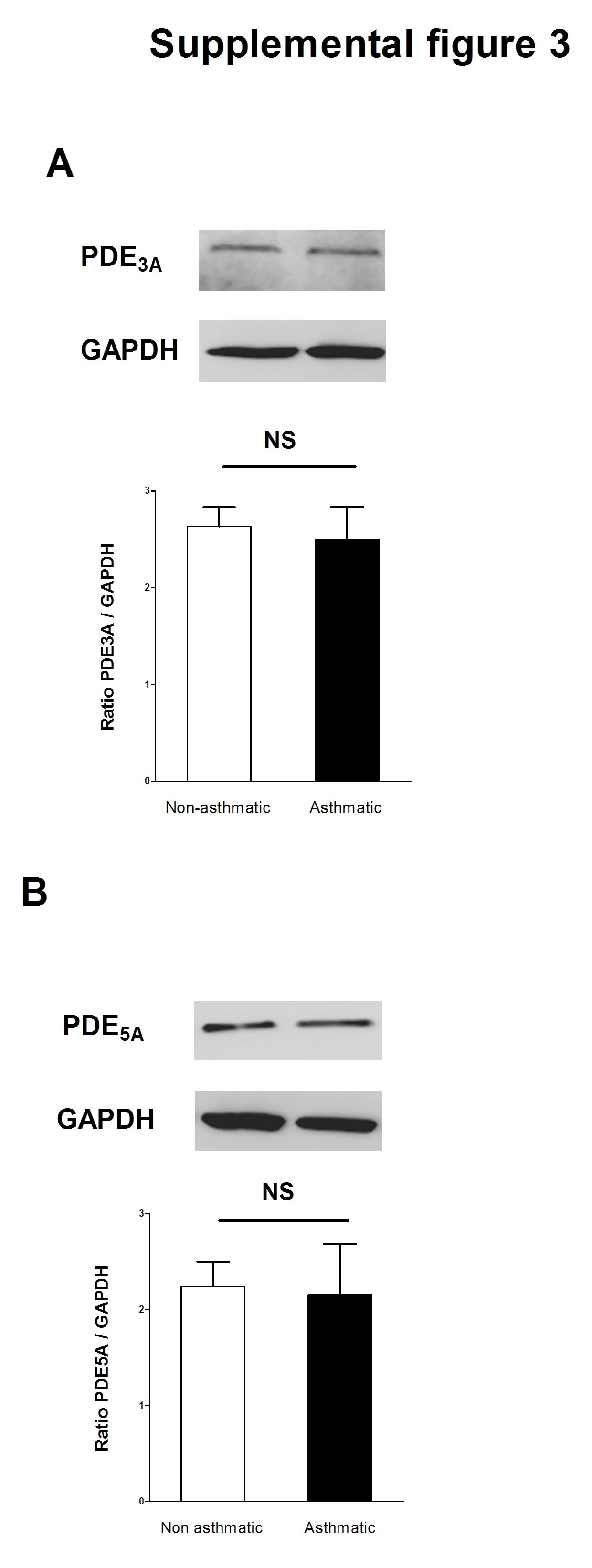

Supplement: Figure S3 — Western blot analysis of A: PDE3B (n = 4 non-asthmatic and n = 4 asthmatic) and B: PDE5A (n = 4 non-asthmatic and n = 4 asthmatic) expression in non-asthmatic and asthmatic ASM cells. A typical western blot is shown for each PDE and the data are summarized in the graph as PDE/GAPDH ratio mean ± SEM. The bands on the left are from non asthmatic, and on the right from asthmatic patients. NS = no significant difference. (TIF) [file pone.0020000.s003.tif]
