# Supplementary material for: β2-Agonist Induced cAMP Is Decreased in Asthmatic Airway Smooth Muscle Due to Increased PDE4D
Source: PLoS One. 2011 May 17;6(5):e20000. doi: 10.1371/journal.pone.0020000 (PMC3096656; doi:10.1371/journal.pone.0020000)
Supplement: Table S1 — Demographics of patient data. (DOCX) [file pone.0020000.s004.docx]

|  |  |  |  |  | |  |  |
| --- | --- | --- | --- | --- | --- | --- | --- |
|  |  | |  |  |  |  |  |
|  | Diagnosis | | Smoking | Gender | age | FEV1 | FVC |
| 1 | Asthma | | N | M | 71 | 57 | 61 |
| 2 | Asthma | | N | M | 45 | 4 | 5.36 |
| 3 | Asthma | | N | M | 71 | 57 | 61 |
| 4 | Asthma | | - | M | 39 | - | - |
| 5 | Asthma | | N | F | 59 | 89 | 93 |
| 6 | Asthma | | Ex | M | 69 | - | - |
| 7 | Asthma | | - | M | 23 | 82 | 81 |
| 8 | Asthma | | - | F | 27 | 79 | 98 |
| 9 | Asthma | | No | M | 23 | - | - |
| 10 | Asthma | | No | F | 61 | 81.00 | 92 |
| 11 | Asthma | | No | M | 21 | 70.00 | 92 |
| 12 | Asthma | | No | F | 40 | 84.2 | 87.8 |
| 13 | Asthma | | - | M | 23 | 82 | 81 |
| 1 | Adenocarcinoma | | N | F | 65 | 87 | 76 |
| 2 | Carcinoma and kyphosis scoliosis | | No | F | 75 | 61 | 58 |
| 3 | carcinoma | | No | M | 79 | 105.5 | 91.4 |
| 4 | carcinoma | | No | F | 77 | 98.9 | 111.6 |
| 5 | Emphysema | | - | M | 47 | - | - |
| 6 | Emphysema | | - | F | 43 | - | - |
| 7 | Bronchiectasis | | N | M | 49 | - | - |
| 8 | Bronchiectasis | | N | - | - | - | - |
| 9 | Small cell carcinoma | | Ex | M | 76 | 46 | 49 |
| 10 | Pulmonary fibrosis | | - | - | - | - | - |
| 11 | emphysema | | - | M | 53 | - | - |
| 12 | bronchictasis | | - | M | 49 | - | - |
| 13 | carcinoma | | Ex | F | 66 | - | - |
| 14 | sarcoiosis | | - | M | 57 | - | - |
| 15 | emphysema | | Yes | M | 55 | - | - |
| 16 | carcinoma | | - | F | 70 | - | - |
| 17 | emphysema | | - | M | 59 | - | - |
